# Interpretation of genotype-environment-sowing date/plant density interaction in sorghum [*Sorghum bicolor* (L.) Moench] in early mature regions of China

**DOI:** 10.3389/fpls.2022.1008198

**Published:** 2022-09-21

**Authors:** Fang-Chao Gao, Hong-Dong Yan, Yue Gao, Yan Huang, Mo Li, Guo-Liang Song, Yue-Mei Ren, Ji-Hong Li, Yan-Xi Jiang, Yu-Jie Tang, Ying-Xia Wang, Tao Liu, Guang-Yu Fan, Zhen-Guo Wang, Rui-Feng Guo, Fan-Hua Meng, Fen-Xia Han, Shao-Jie Jiao, Gui-Ying Li

**Affiliations:** ^1^ National Engineering Laboratory for Crop Molecular Breeding, Institute of Crop Sciences, Chinese Academy of Agricultural Sciences, Beijing, China; ^2^ Crop Resources Institute of Heilongjiang Academy of Agricultural Sciences, Harbin, China; ^3^ Crop Resources Institute of Jilin Academy of Agricultural Sciences, Gongzhuling, China; ^4^ Baicheng Academy of Agricultural Sciences, Baicheng, China; ^5^ Tongliao Institute of Agriculture and Animal Husbandry, Tongliao, China; ^6^ Zhangjiakou Academy of Agricultural Sciences, Zhangjiakou, China; ^7^ High Latitude Crops Institute of Shanxi Agricultural University, Datong, China

**Keywords:** *Sorghum bicolor*, genotype, environment, sowing date, plant density

## Abstract

Sorghum [*Sorghum bicolor* (L.) Moench] is an important crop for food security in semiarid and arid regions due to its high tolerance to abiotic and biotic stresses and its good performance in marginal lands with relatively low fertility. To deeply understand the interrelationship among sorghum genotype, environment, sowing dates, and densities in the spring sowing early maturing (SSEM) areas of China, and to provide a basis for specifying scientific and reasonable cultural practices, a two-year field experiment was conducted with six popular varieties at six locations. Combined ANOVA showed that the yield difference between years was significant (*P<0.05*); the yield differences among locations, varieties, sowing dates, and densities were all highly significant (*P<0.01*). The variety effect was mainly influenced by location, year, sowing dates and their interactions. The sowing effect was mainly influenced by the location, year, variety and their interactions The plant density effect was significantly influenced by location and location-year interaction. Of the contributions of various test factors to yield variance, the location was the largest one (38.18%), followed by variety (12.31%), sowing date (1.53%), density (0.54%), and year (0.09%), with all these single factors accounting for 52.65%. The total contribution of all two-factor interactions accounted for 14.24%, among which the greatest contributor was location-hybrid interaction (8.07%). The total contribution of all three-factor interactions accounted for 14.58%, of which year-location-hybrid interaction was the largest contributor (9.02%). Sowing dates significantly affected model of sorghum growth and development, especially during the late period. The key climatic factors affecting yield were different among the six locations. Weather factors during the grain filling stages contributed much more than those during the early stage to grain yield. Mid-maturing varieties are recommended other than early maturing varieties for the SSEM areas even when late sowing occurs. Sowing as early as possible is recommended for areas with very short frost-free period (Harbin, Tongliao, and Datong). Proper delayed sowing is recommended for areas with a relative long frost-free period (Gongzhuling, Baicheng and Zhangjiakou). This research will provide a conducive reference for sorghum production in similar areas.

## 1 Introduction

Sorghum [*Sorghum bicolor* (L.) Moench] is an important crop for food security in semiarid and arid regions due to its high tolerance to abiotic and biotic stresses and its good performance in marginal lands with relatively low fertility (Prasad and Staggenborg, 2011). In recent years (2016-2020), sorghum was produced averagely on 41.33 million hectares with an average yield of 1439.8 kg ha^-1^ worldwide (FAO, 2022). Highly efficient production requires understanding of sorghum response under diverse management practices and genotype-environment-management (G-E-M) interactions, which would allow optimizing the use of all environment-plant resources and then closing yield gaps (Ciampitti et al., 2019).

Significant yield differences among genotypes and among environments have been observed in almost all studies, and their interaction is often found to contribute a great proportion to the total yield variation (Alagarswamy and Chandra, 1998; Chapman et al., 2000; Carcedo et al., 2021). For example, in Alagarswamy and Chandra (1998)’ pattern analysis of international sorghum multi-environment trials for grain-yield adaptation, a highly significant difference was found among genotypes, the average grain yield of genotypes across environments varied from 1.53 t ha^-1^ to 3.65 t ha^-1^. Yield difference among environments was much larger than that of genotype with the mean grain-yield across genotypes varying from 0.28 t ha^-1^ to 6.23 t ha^-1^. The mean grain-yield in the GE data matrix ranged from 0 to 9.31 t ha^-1^. Genotypes, environments, and GE interactions explained respectively 12%, 61%, and 27% of the total variation, which meant environments accounted for maximum variation, followed by GE interaction and genotypes. Many similar findings were also documented in other GE studies (Rathore et al., 2012; Rakshit et al., 2017; Carcedo et al., 2021). So, it is of significance not only to select a proper variety for a specific region but also to specify a set of favorable production practices.

Planting date has always been a critical aspect of sorghum production practices, which will determine the growth environment. The planting window for maximizing sorghum productivity under diverse environments can assist in closing the yield gap between the maximum potential yield and the yield attainable in each region, so it has been considered important since ever before (Stickler and Pauli, 1961; Conley & Wiebold, 2003; Carcedo et al., 2021). Traditionally, the choice of sowing date has been determined under the consideration of avoiding high temperatures and drought conditions during critical growth stages (Haile & Hofsvang, 2001; Ekeleme et al., 2011) or alleviating damage from other stresses such as disease, insects, or weeds (Gao et al., 2017; Ciampitti et al., 2019). Prasad et al. (2015) found that the critical period susceptible to heat for yield formation was 10 d before and 5 d after flowering and the planting date is a critical management means of regulating flowering time. When these adverse conditions occur during flowering or grain filling, grain yields might decrease significantly because of significant floret abortion, lower seed setting rate, and decreased seed size (Downes, 1972; Saeed and Francis, 1984).

The optimal planting dates for grain sorghum varied with regions, seasons and cropping systems. In regions with high accumulative temperatures, the sowing window is long for variety with different maturity, but the yield varies with different sowing dates. For example, in the largest-scale sowing experiments conducted at Katherine, Australia, it was found that the yields for December sowings were higher than for January sowings, and there was a steady decline with February sowings (Stern, 1968). Experiments, conducted at Lahoma and Efaw of Oklahoma, showed that planting date significantly affected grain sorghum yield. Optimum yields were found when planting in April or May, depending on year, and cooler soil temperatures can limit yield when grain sorghum is planted too early (Zander et al., 2021). However, research in Missouri indicated that planting date had a small and inconsistent effect on grain sorghum yield, and a rather wide planting date window was found (Conley and Wiebold, 2003) . In regions with limited accumulative temperature, such as Ukraine and early mature sorghum zones in China, the sowing window is relative narrow, and the sowing dates are more important for successful seedling establishment and efficient use of light and thermal resources. Research at Lugansk, Ukraine, showed that it was the more profitable to sow sorghum earlier on April 25 than on May 15, a generally accepted date (Baranovsky et al., 2020). In regions where rainfall is a limiting factor, the right sowing dates may mean the efficient use of rainfall resources. A field experiment conducted during the rainy seasons of 2014-2016 at Surat, India, showed that sowing at the time of onset of the monsoon resulted in more grain than 15-45 days late sowing (Saini et al., 2018). Sowing date experiments conducted in 2010 and 2011 at Giza Governorate, Egypt showed that the growth and yield of grain sorghum were significantly affected by sowing date and genotypes. Delayed sowing from April to late May or June resulted in increased growth (El-Raoufetal., 2013) .

Plant density is influential during the early stages of crop growth as it determines the amount of leaf area available for maximum interception of radiation, which is directly related to the photosynthesis product. In sorghum, the yield response to plant density is not as consistent as that in other crops such as maize, because of the tiller-producing ability of sorghum to compensate for low plant density (Berenguer and Faci, 2001; Lafarge and Hammer, 2002). The yield response to plant density may depend on locations, years, row configuration, variety, or other factors, but there is a trend of yield improvement with increasing plant density. In the United States, Welch et al. (1966) concluded that the optimal plant density for increasing yields in dryland systems of Texas was approximately 100,000 to 150,000 plants ha^-1^ (Fischer and Wilson, 1975). Research in North Dakota indicated that the highest grain yields could be expected with plant populations of 172,974-222,395 plant ha^-1^ (Schatz et al., 1990). In Missouri, Conley et al. (2005) found a yield-density linear-plateau response, with yields improved from 6.3 to 7.3 t ha^-1^ when the plant density doubled from 73,600 to 147,300 plants ha^-1^. Conversely, in Texas, Fernandez et al. (2012) did not find any differences in yield when the plant density ranged from 124,000 to 235,000 plants ha^-1^. Schnell et al. (2014) did not observe significant yield differences among different plant densities from 69,000–245,000 plants ha^-1^ across five locations in central Texas. In Australia, research showed that the highest grain yields could be obtained with plant densities of 50,000-100,000 plants ha^-1^ under rainfed conditions with the stability of sorghum grain yield over a wide range of plant densities and crop maturities (Wade and Douglas, 1990). In Iran, an experiment in 2003 showed that the local cultivar Saravan at a density of 260,000 plant ha^-1^ had the highest grain yield among three densities (100,000, 180,000, and 260,000 plant ha^-1^) (Javadi et al., 2006). In Ethiopia, grain yields increased in linear responses to increases in population density from 29,629 to 166,666 plants ha^-1^, and conventional plant density (88,888 plants ha^-1^) is not optimum (Rezazadeh et al., 2019) . In China, research and practices were summarized as factors of determining plant density, which included regions, hybrid and soil fertility. The suitable density was recommended for major sorghum production areas (LAAS, 1988). For example, for the SSEM zone, 150,000 plants ha^-1^ for mid-mature mid-high stalk sorghum variety in high fertile soil; 105,000-120,000 plants ha^-1^ in mid-fertile soil; for early mature dwarf variety, 225,000-300,000 plants ha^-1^ was recommended. These recommendations are still proven reasonable in recent research (Wang et al., 2019; Duan et al., 2019; Han et al., 2021).

The interactions between genotype, and management or between different management practices, such as Genotype-Sowing interaction (Stickler and Pauli, 1961; Hume & Kebede, 1981), Genotype-Density interaction (Ogunlela and Okoh, 1989; Javadi et al., 2006), Density-Fertilization interaction (Dembele et al., 2020; Dembele et al., 2021; Yang et al., 2021), and Density-Irrigation interaction (Alderfasi et al., 2016) are widely studied in sorghum production, far from, however, the relative contribution of each factor and their interactions that influence sorghum yield are well understood (Ciampitti et al., 2019).

Sorghum is an important dryland cereal crop in China and is now an important and essential feedstock in Chinese liquor breweries (Zhou et al., 2017; Cao et al., 2020). Although its distribution in China is very wide, sorghum production is dominantly located in the northern and northeastern parts of the country, where they were designated the spring-sown early maturing (SSEM) zone and spring-sown late maturing zone, respectively (LAAS, 1988). SSEM zone, mainly including the provinces of Heilongjiang and Jilin and the Inner Mongolia Autonomous Region, is the largest grain sorghum production area. This area is characterized by low temperature in early spring, short frost-free days, and narrowed sowing windows as compared with other sorghum planting areas. In the past, sowing dates were set based on more experience than scientific research because minor crops are paid too little attendance, and systematic research has seldom been done on the timing of sowing, especially the interaction of variety-environment-sowing dates. Under the background of changing climate and mechanical agricultural production, tolerance and adaptation to plant density in sorghum are more important as compared with traditional production systems (man-handed thinning to appropriate density) due to its vulnerable seedling establishment. How plant density interacts with sowing dates is also not well understood. The objectives of this study are to: (1) unravel the role of and interaction among genotype, location and management practices (sowing dates and plant density) in SSEM zone of China; (2) identify the key climatic factors leading to the grain yield difference, and to provide a basis for specifying scientific and reasonable cultural practices.

## 2 Materials and methods

### 2.1 Experimental materials

Six varieties mainly popular in SSEM Regions of China were selected as experimental materials, i.e., Longza 10 (LZ10), Longza 22 (LZ22), Jiza 124 (JZ124), Fengza 4 (FZ4), Jinza 22 (JZ22) and Tongza 108 (TZ108). LZ 10 and LZ 22 are early maturing dwarf hybrids (117-136 cm, 115-117 d); JZ124, FZ4,TZ108 and JZ22 are mid-height, mid-maturing hybrids (169-194cm, 119-121 d). Detailed information on these hybrids is showed in **Supplementary Table S1**.

### 2.2 Locations, key soil, and meteorological information

**Figure 1:**
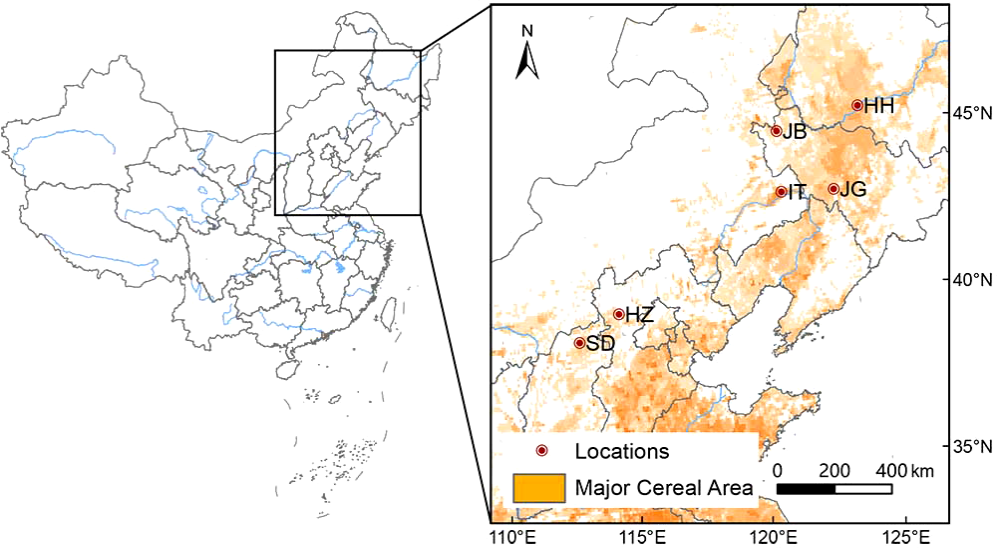
Locations of the study stations. HH, Harbin of Heilongjiang; JB, Baicheng of Jilin; JG, Gongzhuling of Jilin; IT, Tongliao of inner Mongolia; HZ, Zhangjiakou of Hebei; SD, Datong of Shanxi.

Six locations were selected as experimental sites according to their ecological characteristics, i.e., Harbin in Heilongjiang Province (HH), Gongzhuling (JG) and Baicheng (JB) in Jilin Province, Tongliao in Inner Mongolia Autonomous Region (IT), Zhangjiakou in Hebei Province (HZ) and Datong in Shanxi province (SD) (as shown in **Figure 1**). Key meteorological information during the growth period and soil nutrients of 6 locations from 2020-2021 are listed in **Supplementary Tables S2** and **S3**.

### 2.3 Experimental design and management

The field experiment was conducted from 2020 to 2021. Sowing dates were set with local practice as a reference: (1) 10-12 days earlier than the local conventional sowing date (S1); (2) the local conventional sowing date (S2, CK), and (3) 10-12 days later than CK (S3). The density/plant spacing was set as 120,000 plants ha^-1^ (conventional) and 180,000 plants ha^-1^ (high density). A split plot experimental design with three replicates was adopted in this experiment with sowing dates as the main plot, varieties as the split-plot, and density as the sub-split-plot. The plot were 6 rows with 5 meters long row spacing 60 cm, plot area 18 m^2^. The seedlings were manly thinned at the three-leaf stage and settled to designed density at the five-leaf stage. The plots were irrigated before sowing to ensure seedling emergence and then rainfed unless there was serious drought. Diammonium phosphate (150 kg ha^-1^) and potassium sulfate 150 kg ha^-1^) were applied before sowing, urea (300kg ha^-1^) was top-dressed at 7-8 leaf stage. The weeds were controlled with sorghum-special herbicides (mainly containing atrazine and 3,7-dichloroquinoline-8-carboxylic acid).

### 2.4 Data collection and processing

The middle two lines of each plot were harvested for yield estimation. Grain moisture content was determined using a PM-8188A grain moisture analyzer (Tokyo, Japan) and measured 10 times for each kernel sample and recorded the average moisture value. The grain yield was corrected to a standard water content of 14% for the final sorghum grain yield. Important phenological dates such as sowing, seedling emergence, elongation, blooming, and maturity were recorded. Soils samples were collected before fertilization. Data sorting was performed with Excel (2017), and statistical analysis was run with DPS software (Tang & Zhang, 2013). The GGEBiplot installation package was used for the analysis of the relationship between genotype (variety), locations, and years based on the R language. The steps were mainly to calculate the two-way yield analysis of the pilot and variety composition to obtain the environmental centralization formula, and then performed 2 singular value decomposition calculations to obtain principal component 1 and principal component 2 with high scores. Finally, the corresponding biplots were drawn as needed (Jiang et al., 2022). The relative contribution rate (%) of each factor to yield was expressed as a percentage of variance from each factor to the total variance.

## 3 Results

### 3.1 Analysis of variance (ANOVA)

A combined ANOVA was performed for the five factors (year, location, variety, sowing date, and density), as shown in **Table 1**. The results showed that the yield difference between years was significant (*P>0.05*), and the yield differences among locations, varieties, sowing dates and densities were all highly significant (*P>0.01*). The interactions of year with location, sowing dates and year-hybrid were highly significant (*P>0.01*), while not significant with plant density, which showed that the effects of variety, location, and sowing date were different from year to year; the effect of density was similar from year to year. The interactions of location with variety, sowing date, and density were highly significant, indicating that the effects of variety, sowing date and density were all influenced by location. The sowing-variety interaction was significant (*P>0.05*), showing that different varieties responded differently to sowing dates. The interactions of plant density with sowing date and variety were not significant, showing proper high density increased yield for most variety under different sowing dates. Analysis on the three factors interactions showed that the year-location interaction affected the effects of variety, sowing date, and density, while the effect of the sowing date was influenced by the interaction of location-hybrid, or a higher yield could be achieved in a specific location in a specific variety when sowing at the right time. Analysis on the four factors interactions showed that year, location, sowing and variety collectively determined the grain yield, while plant density did not influence the other interaction.

**Table 1:** Combined ANOVA of multiple locations over two years.

Source of variance	SS	Df	MS	F value	*p*-value	Contribution %
Year (Y)	30946.0530	1	30946.0530	5.1876	0.0230	0.09
Location (L)	12785970.4300	5	2557194.0866	428.6704	0.0000	38.18
Sowing dates(S)	511464.9067	2	255732.4533	42.8692	0.0000	1.53
Hybrid(V)	4122514.7830	5	824502.9566	138.2140	0.0000	12.31
Density (D)	181068.9197	1	181068.9197	30.3531	0.0000	0.54
Y×L	519686.2914	5	103937.2583	17.4233	0.0000	1.55
Y×S	57774.58224	2	28887.2911	4.8425	0.0081	0.17
Y×V	477763.2593	5	95552.6519	16.0178	0.0000	1.43
Y×D	3792.661122	1	3792.6611	0.6358	0.4255	0.01
L×S	484125.5116	10	48412.5512	8.1155	0.0000	1.45
L×V	2701357.5500	25	108054.3020	18.1135	0.0000	8.07
L×D	342330.2811	5	68466.0562	11.4772	0.0000	1.02
S×V	134747.9522	10	13474.7952	2.2588	0.0132	0.40
S×D	19289.24474	2	9644.6224	1.6168	0.1991	0.06
V×D	26834.7082	5	5366.9416	0.8997	0.4806	0.08
Y×L×S	765471.1850	10	76547.1185	12.8318	0.0000	2.29
Y×L×V	3021315.3510	25	120852.6140	20.2589	0.0000	9.02
Y×L×D	198751.4096	5	39750.2819	6.6635	0.0000	0.59
Y×S×V	86405.73309	10	8640.5733	1.4484	0.1542	0.26
Y×S×D	12991.52802	2	6495.7640	1.0889	0.3370	0.04
Y×V×D	39318.77685	5	7863.7554	1.3182	0.2540	0.12
L×S×V	510575.0078	50	10211.5002	1.7118	0.0019	1.52
L×S×D	55448.93593	10	5544.8936	0.9295	0.5050	0.17
L×V×D	142595.908	25	5703.8363	0.9562	0.5257	0.43
S×V×D	46533.18885	10	4653.3189	0.7801	0.6482	0.14
Y×L×S×V	421798.2914	50	8435.9658	1.4141	0.0334	1.26
Y×L×S×D	38852.3187	10	3885.2319	0.6513	0.7700	0.12
Y×L×V×D	141287.2086	25	5651.4883	0.9474	0.5382	0.42
Y×S×V×D	16474.03691	10	1647.4037	0.2762	0.9863	0.05
L×S×V×D	259155.9561	50	5183.1191	0.8689	0.7281	0.77
Y×L×S×V×D	179980.4471	50	3599.6089	0.6034	0.9867	0.54
Error	5154113.477	864	5965.4091			
Total	33490735.9	1295				

SS, sum of square; MS, mean square, degree of freedom; Y, year; L, location; S, sowing date; G, genotype(variety); Densit**y**.

From the contribution of various test factors and their interaction to yield variation, within the scope of this study, the location contribution rate was the largest (38.18%), followed by varieties (12.31%), sowing date (1.53%) and density (0.54%), the year contribution rate was relatively small, accounting for only 0.09%, all these single factors accounting for 52.65% of the total yield variance. The total contribution rate of all two-factor interactions accounted for 14.24%, among which the interactions with greater contribution rates were location-variety (8.07%), followed by year-location (1.55%), location-sowing (1.45%), year-variety (1.43%) and location-density (1.02%), showing that the selection of a specific variety for a given location was a prerequire for high-yield production. For some locations, density and sowing dates were important. The total contribution rate of all three-factor interactions accounted for 14.58%, of which the larger contribution rates were from year-location-variety (9.02%), year-location-sowing (2.29%) and location-sowing-variety (1.52%), all involved in locations; the total contribution rate of the 4-factor interaction was only 2.62%, only year-location-sowing-variety reaching a significant level. The contribution rate of the 5-factor interaction was only 0.54%.

To further interpret the variety-sowing date-plant density in each location, ANOVAs were also performed location by location. The significance of each factor and interaction (shown with *P* values) was listed in **Table 2**. The results showed that the effects of sowing dates were different from location to location, and from year to year. For example, at Gongzhuling (JG), the effect of the sowing date approached a significant level (*P=0.0507*) in 2020 and was not significant in 2021. At Tongliao (IT), the sowing date effect was not significant but was significant in 2021; At Zhangjiakou (HZ), it was not significant in two years; At Datong (SD), it approached significance in 2020 and was significant in 2021; and at Baicheng (JB), it was significant in two years. At Harbin (HH), it was significant or nearly significant in two years. The yield difference among varieties was highly significant in all locations in the two years. The effects of plant density also varied by location and year. For example, it was significant in two years at HZ; significant in one year at JG and SD; and not significant at IT, JB and HH in two years.

**Table 2:** Significant *P* value of ANOVA in different locations and years.

Location		JG		IT		HZ		SD		JB		HH
Source of Variance	2020	2021	2020	2021	2020	2021	2020	2021	2020	2021	2020	2021
Variety(V)	0.0001	0.0001	0.0000	0.0010	0.0001	0.0000	0.0001	0.0001	0.0000	0.0001	0.0000	0.0001
Sowing dates (S)	0.0507	0.1994	0.2003	0.0060	0.1255	0.4741	0.0867	0.0022	0.0093	0.013	0.0435	0.0888
Density(D)	0.0058	0.4183	0.9198	0.6647	0.0333	0.0000	0.7341	0.0147	0.3188	0.9316	0.4116	0.2177
V×S	0.2277	0.9765	0.3306	0.0504	0.1266	0.1095	0.0025	0.0001	0.1459	0.1146	0.0018	0.0386
V×D	0.2911	0.4801	0.1043	0.5545	0.5806	0.02646	0.7301	0.3288	0.9635	0.0296	0.6113	0.2224
S× D	0.7902	0.6615	0.3081	0.0569	0.2667	0.61176	0.4571	0.1472	0.6634	0.7465	0.6652	0.1434
S×-V×D	0.3642	0.8192	0.8125	0.8690	0.9913	0.78685	0.8743	0.6750	0.7518	0.0779	0.1469	0.5827

JG, Gongzhuling, Jilin; IT, Tongliao, inner Mongolia; HZ, Zhangjiakou, Hebei; SD, Datong, Shanxi; JB, Baicheng, Jilin; HH, Harbin, Heilongjiang. V, Variety; S, Sowing dates; D, Plant density.

### 3.2 Mean performance of test varieties

The yield performance of the six varieties varied with location, year, sowing date, and plant density, as shown in **Table 3** and **Supplementary Figure S1**. Overall, the variety TZ108 performed best in 2020, and JZ124 performed best in 2021. Variety LZ10 and LZ22 performed similarly in yield, with the lowest yield among the six varieties in the two years. Throughout the whole experiment, the average yield of JZ124 in all cases was the highest, 10.24 t ha^-1^. The yields of JZ22, FZ4, and TZ108 were at the same level, with values of 9.79 t ha^-1^, 9.72 t ha^-1^, and 9.70 t ha^-1^, respectively. The yield difference between these three varieties and JZ 124 was highly significant (*P<0.01*). The yields of LZ 22 and LZ 10 were similar and were significantly lower than those of the other 4 varieties. Given the average yield of different sowing dates and densities, the six varieties performed differently at different locations. TZ108 performed best at 2 locations (JB and HZ) in 2020 and 1 location (IT) in 2021. JZ124 won other varieties in yield at 1 location (JG) in 2020 and 2 locations (SD, HZ) in 2021. FZ4 won at 2 locations (HH, SD) in 2020 and 2 locations (HH, JB) in 2021. JZ22 won at 1 location (IT) in 2020 and 1 location (JG) in 2021.

**Table 3:** Yield performance of six varieties under different sowing dates and dates at six locations in 2020-2021.

Year	Items							
2020	Location	HH	7.33 b	7.44 b	8.76 a	**9.22 a**	8.49 a	8.58 a
		JB	8.24 b	10.80 a	8.59 b	11.00 a	11.13 a	**11.78 a**
		SD	5.64 b	4.96 b	7.38 a	**7.45 a**	7.41 a	7.03 a
		HZ	9.97 b	8.29 c	11.41 a	11.17 ab	11.05 ab	**12.12 a**
		IT	6.83 c	7.45 c	9.47 ab	9.14 b	**10.00 a**	9.47 ab
		JG	11.22 b	11.92 ab	**13.67 a**	8.18 c	8.37 c	10.55 b
	Sowing dates	S1	8.80 cd	8.50 d	9.78 ab	9.70 ab	9.52 bc	**10.35 a**
		S2	8.32 c	8.79 bc	10.22 a	9.67 ab	9.81 a	**10.26 a**
		S3	7.50 d	8.14 cd	**9.63 a**	8.71 bc	8.90 abc	9.16 ab
	Density	D1	8.01 c	8.31 c	9.44 ab	9.22 b	9.26 b	**9.80 a**
		D2	8.40 d	8.65 d	**10.32 a**	9.50 c	9.56 bc	10.04 ab
	Mean-variety		8.21 b	8.48 b	9.88 a	9.36 a	9.41 a	**9.92 a**
2021	Location	HH	7.78 c	8.11 c	9.27 ab	**10.05 a**	8.52 bc	9.05 b
		JB	9.31 c	9.33 c	11.27 ab	**11.74 a**	11.32 ab	11.01 b
		SD	4.28 e	7.37 b	**8.99 a**	6.50 c	7.84 b	5.58 d
		HZ	9.86 c	7.65 d	**13.91 a**	12.93 ab	13.40 a	12.12 b
		IT	7.19 b	8.78 a	9.10 a	8.16 ab	8.69 a	**9.40 a**
		JG	7.50 b	7.70 b	11.04 a	10.82 a	**11.27 a**	9.94 a
	Sowing dates	S1	7.79 c	7.93 c	**10.85 a**	10.31 ab	10.79 ab	10.00 b
		S2	7.68 d	8.46 c	**10.52 a**	9.99 ab	10.18 ab	9.59 b
		S3	7.50 d	8.07 d	**10.42 a**	9.80 ab	9.56 bc	8.96 c
	Density	D1	7.48 e	8.00 d	**10.48 a**	10.08 b	9.99 b	9.20 c
		D2	7.83 e	8.31 d	**10.71 a**	9.99 bc	10.36 ab	9.84 c
	Mean-variety		7.66 e	8.16 d	**10.60 a**	10.03 b	10.17 b	9.52 c

Letters of a, b, c, d, e are used to show the significance of the difference, and the same letter means that the difference is not significant. The number in bold indicates the highest yield among the six varieties under a given treatment.

In the case of different sowing dates, JZ124 won other varieties under late sowing (S3) in 2020, and under all sowing dates in 2021; TZ108 won other varieties under conventional sowing (S2) and early sowing (S1) in 2020. Those results showed that JZ124 may be a better choice when having to sow late. In case of different plant densities, JZ124 performed better under a higher density (D2) in 2020, and under both 2 densities in 2021. TZ108 performed best under normal density (D1) in 2020.

From the above analysis, the grain yield of the early-maturing varieties (LZ10, LZ22) was lower than that of the mid-maturing varieties in almost all cases even when late sowing led to the incomplete maturity of the mid-maturing varieties, which showed that the mid-maturing varieties were of higher yield potential than the early-maturing varieties for the SSEM region.

### 3.3 Effect of locations and years

**Figure 2:**
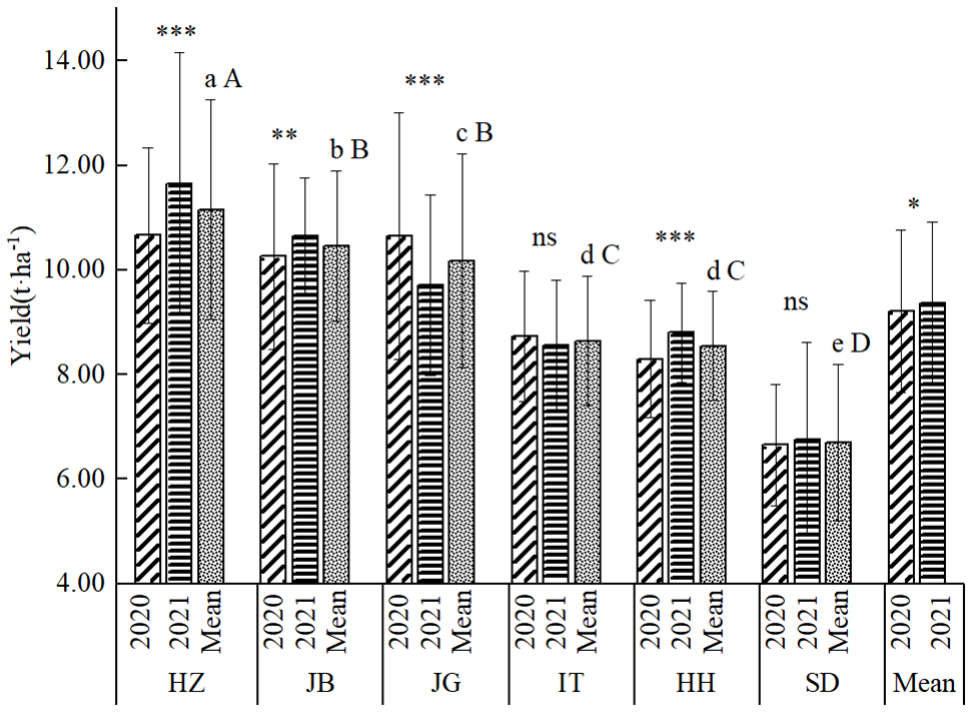
General yield performance at six locations in 2020-2021. a, b, c, d, c, e, **(A–D)**, indicating mean yield difference significance among locations at 0.05, and 0.01; *, ** and *** indicating difference significance between two years at 0.05, 0.01 and 0.001.

As shown in **Figure 2**, there seemed little difference in the average yield between the two years, but it reached a significant level at α=0.05, indicating that there existed a true yield difference. Overall, the average yield in the two years was 9.28 t ha^-1^, of which the average yield in the whole test in 2020 was 9.21 t ha^-1^, and the average yield in 2021 was 9.36 t ha^-1^. The yield difference among locations was highly significant, with the highest yield at HZ, with an average yield of 11.16 t ha^-1^ in two years, followed by JB, with an average yield of 10.46 t ha^-1^; JG, with an average yield of 10.18 t ha^-1^; IT, the average yield of 8.64 t ha^-1^; HH, the average yield of 8.55t ha^-1^; SD, the average yield of 6.65 t ha^-1^. The mean yield of HZ was highly significantly higher than that of JB; The mean yield of JB was significantly higher than that of JG; The mean yield of JG was highly significantly higher than that of IT and HH; there was no significant yield difference between IT and HH, but significantly higher than SD.

**Figure 3:**
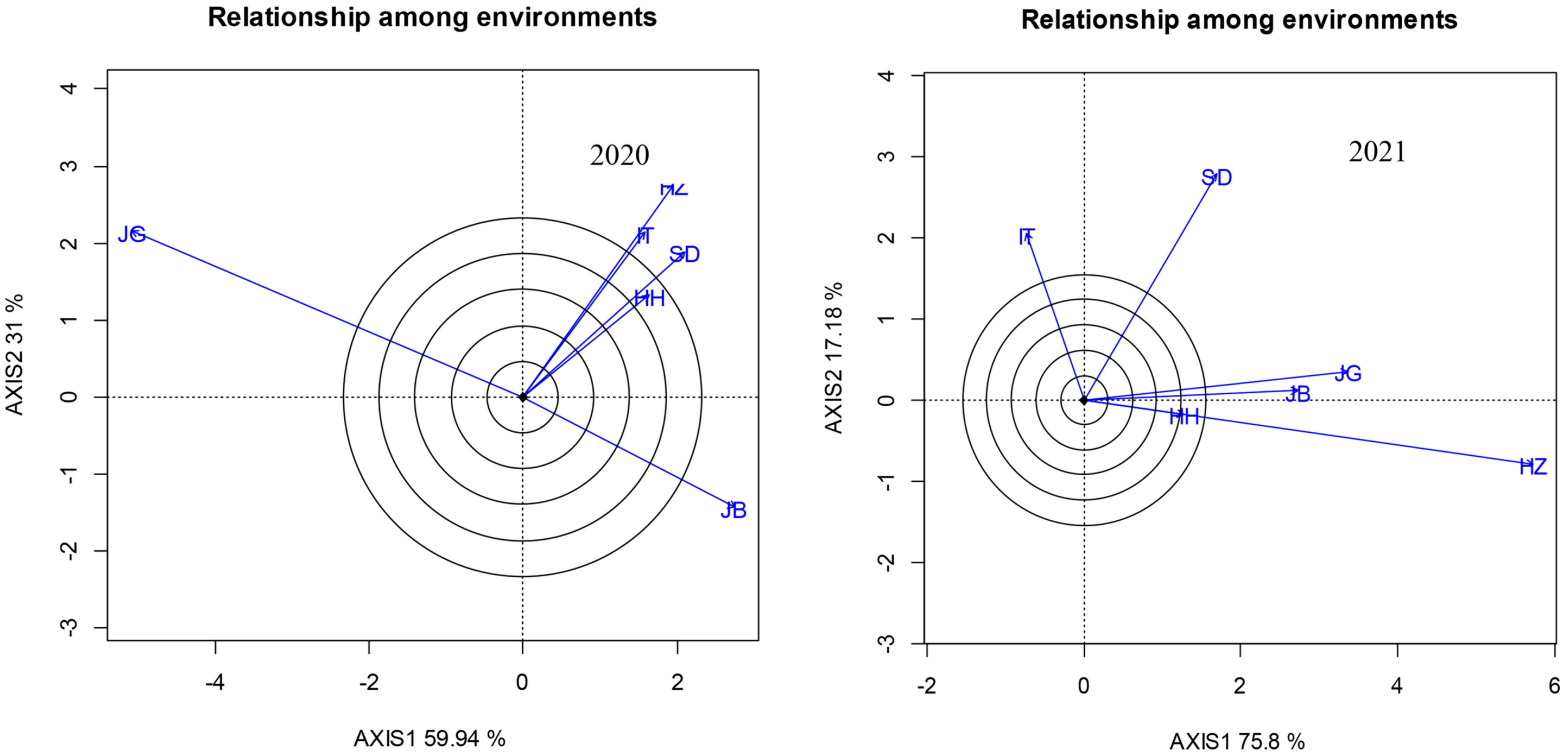
Relationship among locations under different sowing dates revealed by RDA analysis.

**Figure 3** is a biplot of two PCAs to show the relationship among locations revealed by RDA analysis to evaluate the distinction and similarity of each environment. The results showed that in 2020, the angles between JG and the other experimental sites were all greater than 90°, showing a negative correlation existed, while the environmental lines of IT and HZ, SD and HH were close to overlapping, showing that the environments of IT, HZ, SD, and HH in 2020 had a strong similarity. The angles between JB and HZ, IT, SD, and HH were approximately 90°, showing a weak correlation between the two types of environments. At the same time, the long axis of JG also showed that it was the location with the most ability to distinguish varieties in 2020, and the HH environment line was the shortest, indicating that HH had the weakest ability to distinguish varieties, and the yields of varieties under its environment were relatively similar and difficult to distinguish. In the environmental relationship diagram in 2021, HZ showed the strongest ability to distinguish varieties, and HH had the weakest ability to distinguish varieties. At the same time, the environmental lines of that HZ and HH almost overlapped, indicating that the two environments were highly similar in 2021, and HZ and HH were highly similar. The angles between HH/HZ and JG and JB were very small, indicating that the environments of the four sites had greater similarity. Comparison of two years showed great change of JG and JB in ability to distinguish varieties.

**Figure 4:**
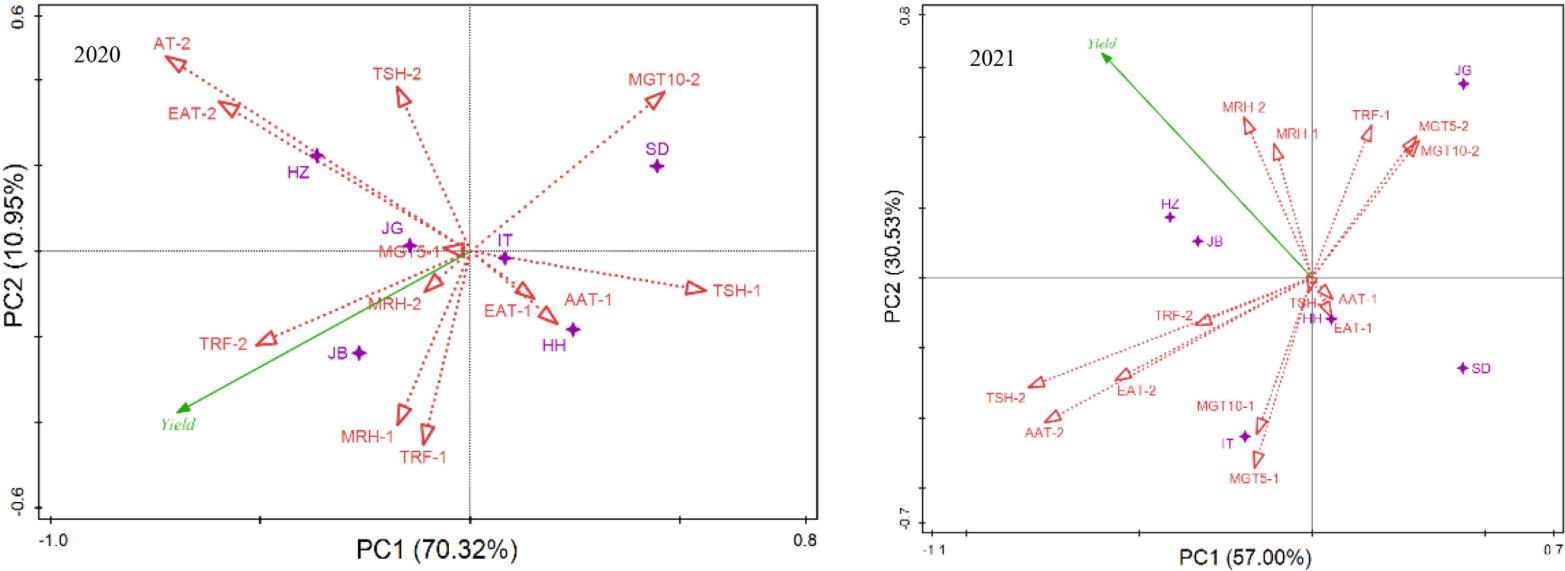
Relationship of location and ecological factors revealed by RDA analysis of yield. EAT, effective accumulative temperature; AAT, accumulative average temperature; AGT5, average 5-cm ground temperature; AGT10, average 10-cm ground temperature; ARH, average relative humidity per day; TRF, total rainfall; TSH, total sunshine. -1, stage from emergence to flowering; -2, stage from flowering to mature.

To understand the key weather factors that resulted in yield differences among locations, RDA analysis was performed, as shown in **Figure 4**. Relationships of climatic factors with yield showed different models at different locations in two years. In 2020, at JB, mean 5-cm ground temperature (MGT5) and mean relative humidity (MRH) from emergence to anthesis and total rainfall (TRF) from anthesis to mature, were the key contributors to the yield; at HZ and JG, temperature (accumulative average temperature, AAT-2 and effective accumulative temperature, EAT-2) and sunlight (total sunshine, TSH-2) during grain-filling were the major contributors. At SD, the ground temperature during the seed filling stage (MGT5-2, MGT10-2) was a key factor; at HH and IT, sunlight and temperature (EAT-1, TSH-1, AAT-1) during the early stage were the key factors. In 2021, at HZ and JB, moisture (TRF-1, MRH-1, MRH-2) and temperature (MGT5-2, MGT10-2) were the key factors; at JG, the key factors affecting the grain yield was the temperature during the late development stage/seed filling stage (AAT-2, EAT-2, AAT-2); at SD and HH, temperature (AAT-1) and sunlight (TSH-1) during the early stage were the key factors; and at IT, ground temperature (MGT5-1, MGT10-1) during the early period was the key factor.

### 3.4 Effect of sowing dates and plant densities

**Figure 5:**
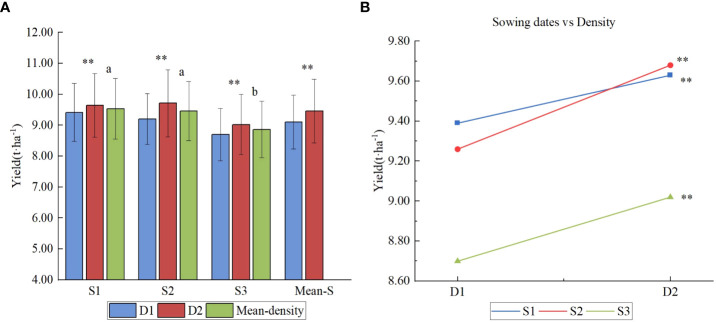
Yield effects of different sowing period and densities. **(A)** Yield of different sowing dates under two densities; **(B)** Interaction of sowing dates and density. Letters a and b are used to label the difference significance at 0.05; the same letter means not significant (NS) among the three sowing dates; ** means highly significant at 0.01 between the two densities.

As shown in **Figure 5A**, the mean yield of sowing date 1 (S1) was the highest, averaged 9.51 t ha^-1^. The mean yield of sowing date 2 (S2) was equivalent to that of sowing date 1 (S1), averaged 9.47 t ha^-1^, which was not significantly different from that of sowing date 1(S1). The mean yield of late sowing (S3) was 8.86 t ha^-1^, which was significantly lower than that of early sowing (S1) and conventional sowing date 2 (S2). Plant density was found to have a significant effect on yield. The mean yield at density 2 was 9.45 t ha^-1^, 3.61% higher than that at density 1, reaching a very significant level (*P<0.05*). **Figure 5B** shows that there was no significant interaction between sowing date and density, which showed that the density could be appropriately increased whether it is sown early or late.

**Figure 6:**
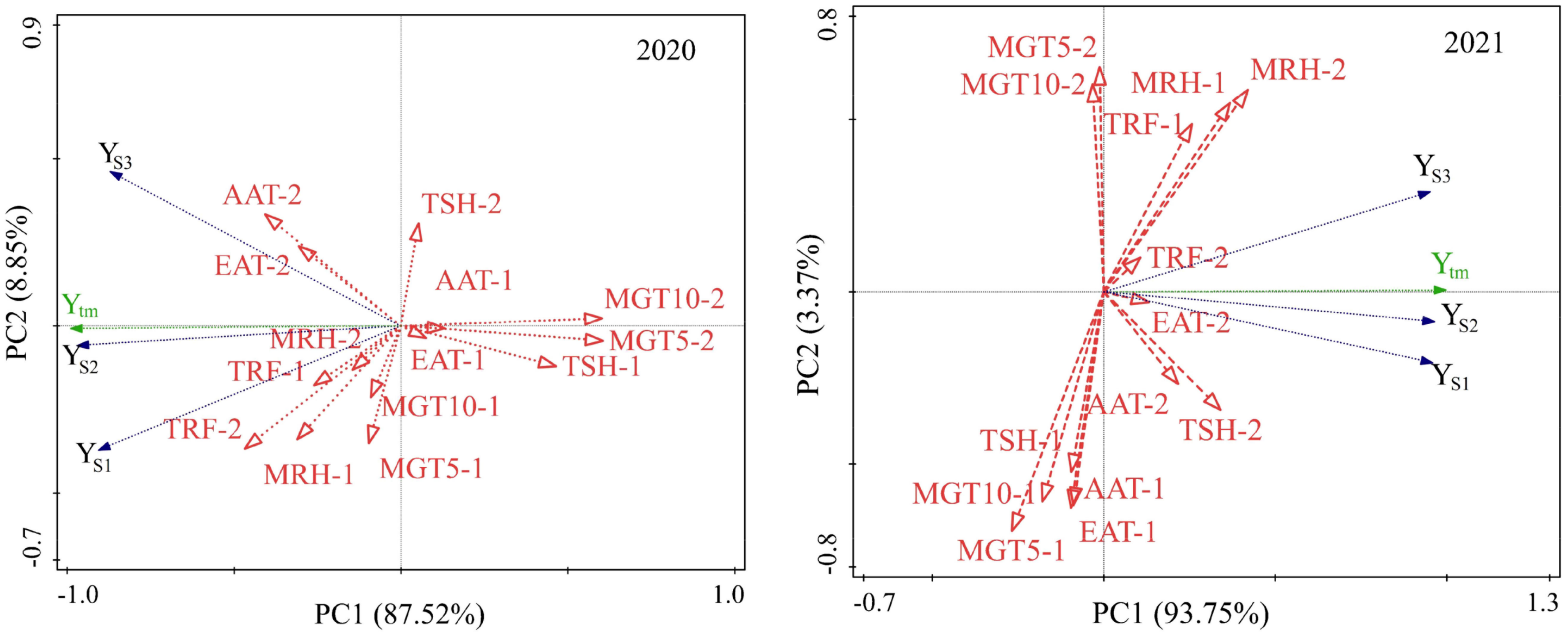
RDA analysis of yields on sowing dates in response to climatic factors during emergence to flowering and flowering to maturity in 2020 (left) and 2021(right). EAT, effective accumulative temperature; AAT, accumulative average temperature; AGT5, average 5-cm ground temperature;AGT10, average 10-cm ground temperature; ARH, average relative humidity per day; TRF, total rainfall; TSH, total sunshine. -1, stage from emergence to flowering; -2, stage from flowering to mature.

Redundancy analysis was performed with the mean yields of different sowing dates in six locations vs climatic factors during two development stages, i.e., stage 1, from emergence to anthesis; and stage 2, from anthesis to maturity. The conditional effects (R^2^ value or % explained) of each explanatory factor were calculated (**Supplementary Tables S4** and **S5**, and biplots were constructed with the two principal components, as shown in **Figure 6**. In 2020 and 2021, climatic factors during the second stage contributed much more proportion to the yield variance, 67.8%, and 79.8% respectively, which showed that the weather played a more important role in yield formation at the grain-filling stage than at the vegetable development stage. Similarly in two years, ground temperature at the seed-filling stage (MGT5-2) contributed most to the yield variation, 32.8% and 23.1% respectively in 2020 and 2021. Sunlight (TSH) at the grain-filling stage (MGT5-2) played an important role in yield formation (*P=0.052*, 2020; *P=0.034*, 2021). In addition, air humidity at the grain-filling stage (MRH-2) also contributed for a great proportion of the total yield variation (17.8%, *P=0.07*) in 2021. As for the vegetable stage, effective accumulative temperature (EAT-1) played significantly important role in 2020 (*P=0.026*); 10cm-ground temperature and mean relative humidity played significantly important role in 2021 (*P=0.046, 0.008*).

The relationship between yields of the three sowings and climatic factors showed a different model in 2020 and 2021(**Figure 6**). In 2020, the mean yield of early sowing was more closely related to moisture factors (MRH-1, MRH-2, TRF-1, TRF-2) than that of the other two sowing dates. There was a positive relation to an extent between the yield at early sowing and ground temperature during stage 1 (MGT5-1, MGT10-1). The mean yield of late sowing was more closely related to temperature factors (AAT-2, EAT-2), and sunlight (TSH-2) than the other two sowings. The accumulative temperature (AAT-1, EAT-1) and sunlight (TSH-1) during stage 1 and ground temperature during stage 2 were negatively related to the yield of all three sowing. In 2021, the yield of early sowing was more closely related to sunlight and temperature factors during stage 2 (TSH-2, AAT-2, EAT-2); the mean yield of late sowing was more closely related to moisture factors (MRH-2, MRH-1, TRF-1 and TRF-2), which showed that temperature was the key factor affecting yield when early sowing, and rainfall was the key factor affecting yield when late sowing. There was an extent of positive relation between yield and ground temperature during stage 2 (MGT5-2, MGT10-2) for late sowing, but no any relation for the other two sowing, which showed ground temperature during late stage had a greater influence on the yield when late sowing than the other two sowing. Temperature and sunlight factors (TSH-1, EAT-1, AAT-1, MGT5-1, and MGT10-1) during the first stage were not significantly or negatively related to yield, showing that climatic conditions of early stage had relatively less effect on yield.

### 3.5 Interaction among variety, year, location, sowing date, and density

As shown in ANOVA (**Table 2**), significant or highly significant interactions were already known among variety, year, location, sowing date, and density. Here, we further interpret in detail the two-factor interactions, including interaction of hybrid with location and year; interaction of sowing year, location and hybrid; interaction of plant density and location, and try to explain three -factor interactions including interactions of year-location-sowing and location-sowing-hybrid.

#### 3.5.1 Interactions of variety with year and location

The six varieties performed significantly differently in the two years (**Supplementary Figure S2**). The varieties JZ124 and JZ22 performed significantly or highly better in 2021 than that in 2020; FZ4 performed better in 2021 than in 2020 but did not reach a significant level at 0.05; the other three varieties TZ108, LZ10 and LZ22 showed significant or highly significant yield decreases in 2021 than that in 2020. Given the great difference in climatic conditions between the two years, this result indicated that different varieties responded significantly to climate change. TZ108, LZ10, and LZ22 were developed at higher latitude area and may adapt better to climates with less high-temperature stress.

**Figure 7:**
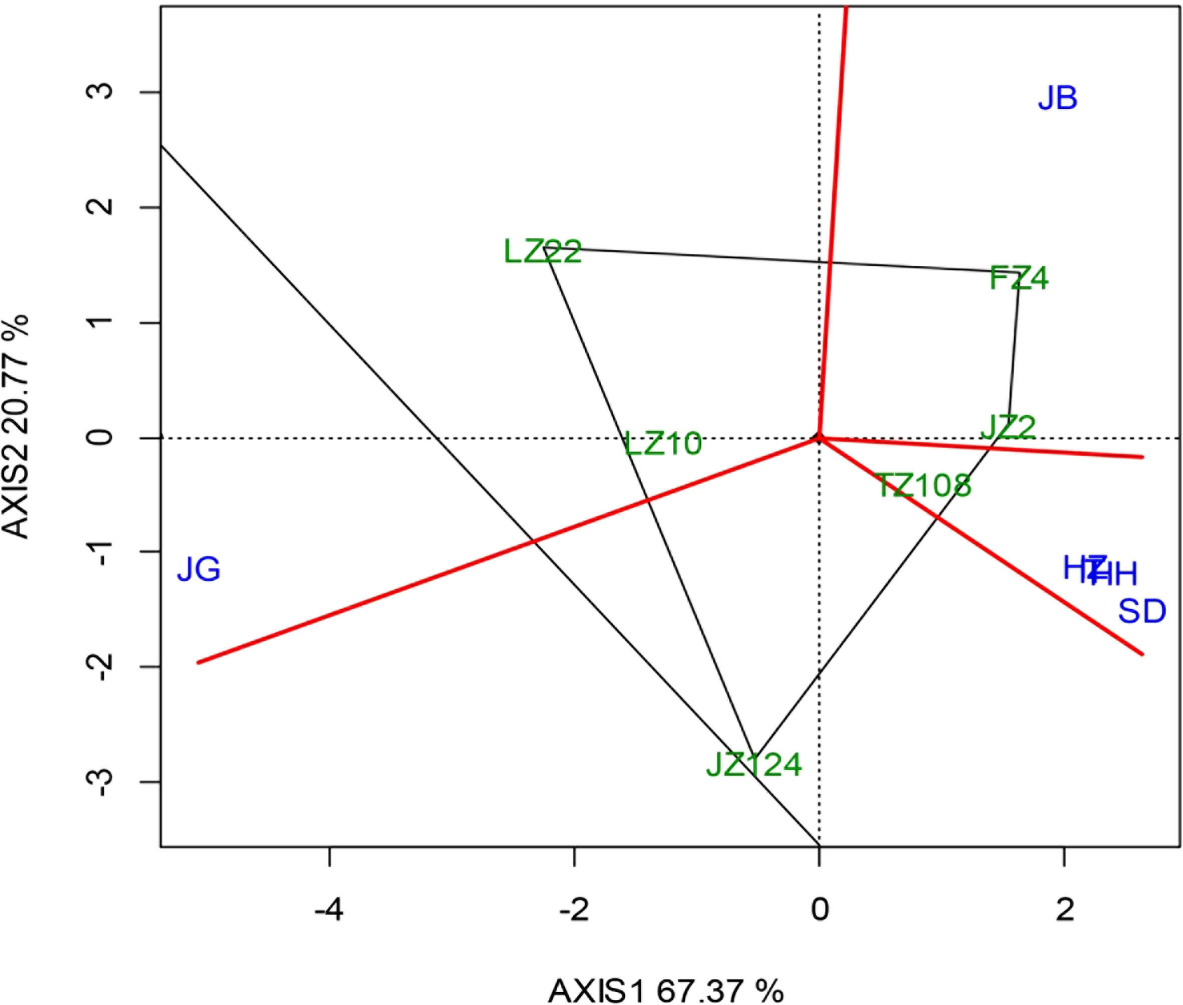
“Which Won Where/What” biplot under different sowing dates.

The AMMI biplot (**Figure 7**) was constructed with the first two principal components that explained 88.14% of the variety-location interaction, in which PCA1 and PCA2 contributed 67.37% and 20.77%, respectively. Under conventional sowing, different varieties performed differently among locations: TZ108 performed well at locations HH, SD, IT, and HZ; JZ22 and FZ4 performed well at JB; LZ10 and LZ22 performed well at JG. The perpendicular projection from variety to location vector reflects the amount of interaction with a given location. The length of the vector of a location from the biplot origin means that it is proportional to the amount of location-variety interaction. It was shown that location JG witnessed the strongest interactive effect, followed by JB and SD, then was HZ, HH and IT, where the interactive effects were very similar. By connecting the varieties farthest from each direction with green straight lines to form a quadrilateral and making four vertical lines (red lines) on the four sides through the center to divide the diagram into four sectors, the six study locations were in three of these sectors. SD, HZ, HH and IT were in one sector, while JB and JG were respectively in the other two sectors. FZ4 had the highest yield in JB, while TZ108 had the lowest yield in SD, HZ, HH, and IT. The hybrid LZ22 performed best at G.

#### 3.5.2 Interactions of sowing dates with variety, year, and location

**Figure 8:**
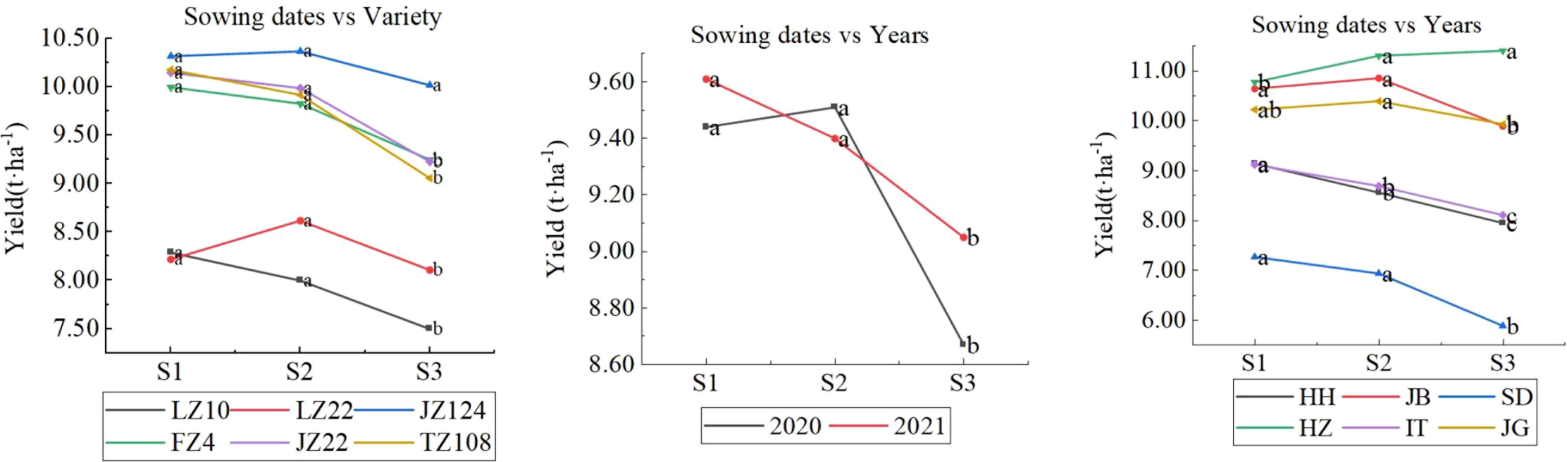
Interactions between sowing dates and variety (left), years (mid), and locations (right). Letters a, b and c are used to label the difference significance at 0.05; the same letter means not significant (NS), and a different letter means significant among the three sowing dates.

As shown in **Figure 8**, the six varieties responded differently to sowing dates. On average, JZ124 exhibited a yield decrease when early sowing or late sowing but did not reach a significant level at 0.05. The varieties FZ4, JZ22, and TZ108 performed similarly in that yield increased slightly when early sowing but not significantly and decreased significantly when late sowing. LZ22 performed yield decreased under both early sowing and late sowing. LZ10, with the lowest yield potential, exhibited a significant yield increase when early sowing occurred, but the increase was not significant and decreased significantly when late sowing occurred. Regarding sowing dates and years, their interaction showed a yield decrease in 2020 and an increase in 2021 when early sowing occurred, although the difference was not significant. In addition, the extent of yield decreases when late sowing in the two years was different, 3.87% in 2020, and 9.69% in 2021. Regarding the interaction of sowing dates and location, six locations responded differently to sowing dates in that yield increased when early sowing(S1) occurred at SD, IT, and HH, which was not significant at SD and was significant at IT and HH; yield didn’t decrease significantly when early sowing occurred at JB and JG; yield decreased significantly when early sowing and yield increased insignificantly.

#### 3.5.3 Plant density-location interaction

As shown in **Supplementary Figure S3**, the effects of plant densities depended on the location in that yield increased significantly at HZ and JG and increased or decreased insignificantly at the other four locations. We may note that HZ and JG had a higher grain yield, which may indicate that a higher density is more suitable for high-yield areas. Year-location interaction is a common phenomenon in agricultural production. In this research, the yield was different between the two years in different locations. Yield increased significantly in 2021 as compared with that in 2020 at HZ, JB, and HH. Yield in 2021 decreased significantly as compared with that in 2020 at JG. At SD and IT, the yield difference between the two years was not significant.

#### 3.5.4 Year-sowing date-location interaction

**Figure 9:**
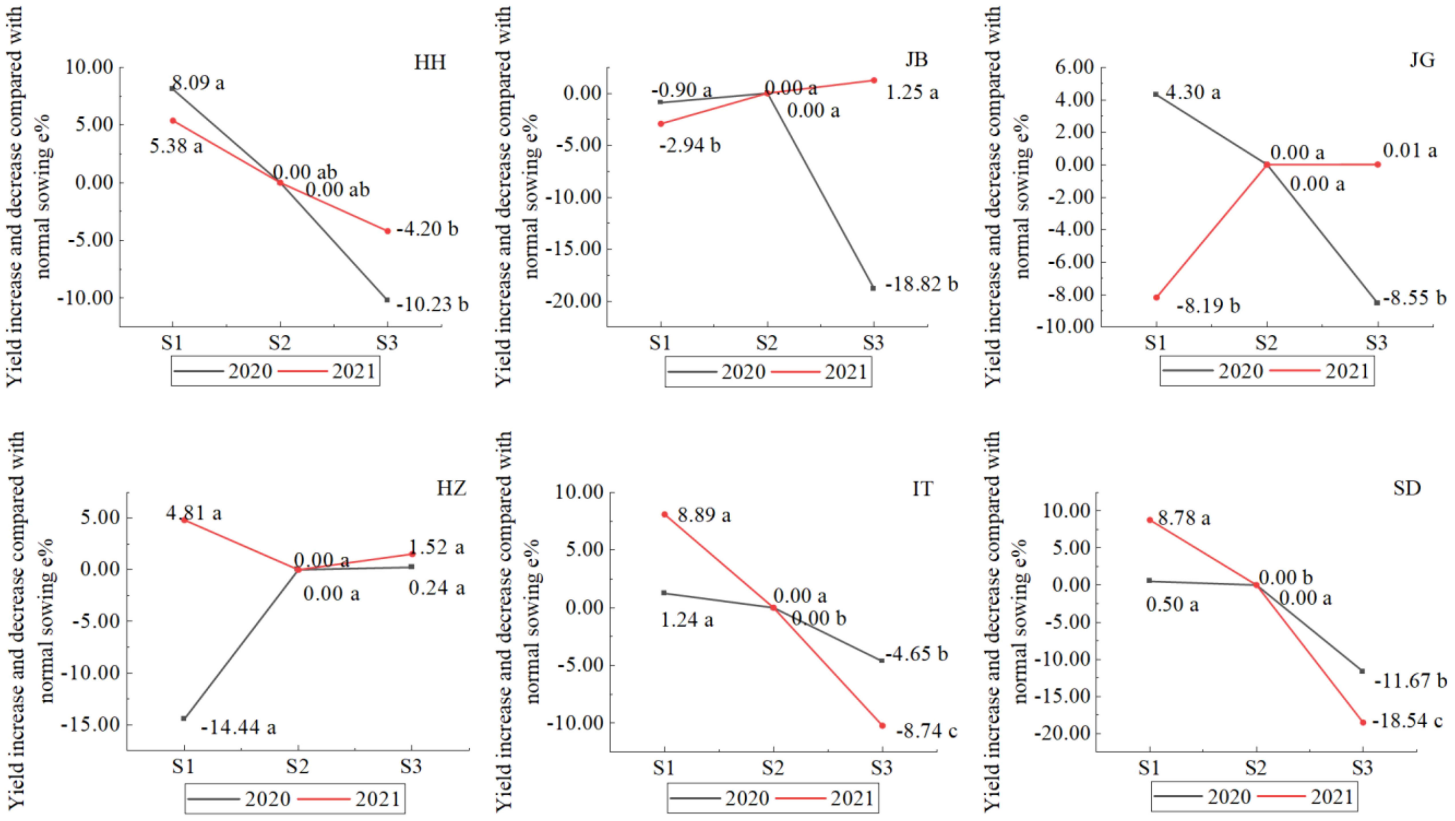
Effect of sowing date on sorghum grain yield in different locations (Letters a–c), after data means difference significance at 0.05, the same letters mean not significant).

As shown in **Figure 9**, the interactive effect of year and sowing date in different locations showed four different models: (1) yield increase when early sowing, decrease when late sowing in two years, like at HH, IT, and SD; (2) yield decrease when early sowing in two years, like in JB; (3) yield increase in one year, and decease in another year when early sowing, and no yield increase or significant when late sowing, like at JG; (4) yield increase in one year, and decease in another year when early sowing, yield increase insignificantly when late sowing, like at HZ. At HH, early sowing showed a yield increase of 5.38-8.09% as compared with normal sowing (S2), but did not reach a significant level at 0.05, and late sowing (S3) showed a yield decrease of 4-2-10.2% and did not reach a significant level at 0.05; however, the yields of late sowings (S3) were significantly lower than those of early sowing (S1). At SD, the effect trend of sowing dates was similar to that at HH; early sowing showed a yield increase of 0.5-8.8%, and late sowing significantly decreased by 11.7-18.5%. HH, SD, and IT showed similar effect trends for sowing dates, and early sowing showed a yield increase by 5.4-8.1%, 1.24-8.89%, and 0.5-8.8%, respectively, in the two years. Late sowing showed decreases of 4.2-10.2, 4.65-8.74, and 11.7-18.5%. At JB, the yield decreased by 0.90-2.94% when early sowing occurred in 2020-2021, which was not significant at 0.05 in 2020, but significant in 2021; the yield of late sowing (S3) decreased by 18.82% in 2020 but increased by 1.25% (not significant at 0.05) in 2021. This result showed that there was a high risk when late sowing if we were not familiar with the change law of climate. At JG, it showed a different sowing effect; early sowing showed an increase of 4.3% in 2020 and a decrease of 8.19% in 2021. At HZ, yield increased by 4.81% in 2020 and decreased by 14.44% in 2021 when early sowing occurred; yield did not increase significantly when late sowing occurred in two years.

#### 3.5.5 Sowing date-year-location interaction responded in sorghum development process

**Figure 10:**
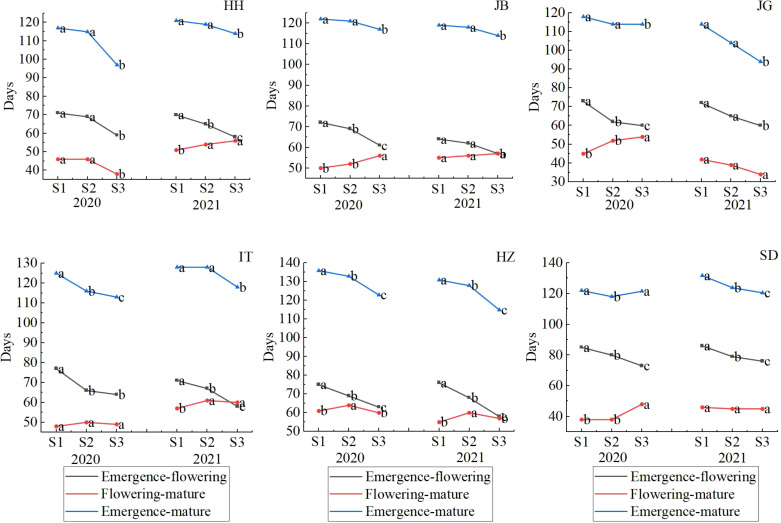
Effects of sowing dates on sorghum development progress in different locations in 2020-2021.Letters (a–c) are used to label the difference significance at 0.05, and the same letter means not significant.

To understand why different sowing dates led to different yields, we analyzed the difference in sorghum development progress when sowing occurred on different dates in different locations, as shown in **Supplementary Figure 4**. It was shown that the responsiveness of all varieties tested to sowing dates showed a similar model, so we analyzed the effect of sowing dates with the mean of all varieties (as showed in **Figure 10**), which showed that days from emergence to maturity generally became shorter as with delayed sowing with one exception, i.e., in SD in 2021, where days from emergence to maturity was 122d when late sowing occurred (S3) and 4 days longer than conventional sowing dates (S2). Changes in days of different stages (emergence to flowering and flowering to mature) were different among locations and between the two years. The days from emergence to flowering regularly decreased as with delayed sowing (from S1-S3). However, the days from flowering to maturity (D_f-m_) changed greatly from year to year, and from location to location. At HH, D_f-m/_S1 was the same as D_f-m_/S2 (46 d), and D_f-m/_S3 shortened to 38 d in 2020, but D_f-m_ became longer as with delayed sowing, from 51 d to 54 d to 56 d in 2021. At JB, D_f-m_ was longer in 2021 (56 d/S2) than that in 2020 (52 d/S2), 2 days earlier when early sowing (S1) than conventional sowing (S2) in 2020, 4 d later when late sowing (S3) than in S2; 1d s earlier when early sowing (S1) and 1 d later when late sowing (S3) than in S2 in 2021. At JG, the model looked the opposite in two years. In 2020, D_f-m_ became longer as with delayed sowing, whereas in 2021, it was the opposite. D_f-m_ was significantly shorter in 2021 (39 d/S2) than in 2020 (52 d/S2). At IT, it looked similar to that at HH with minor differences in those days from emergence-flowering, which prolonged when early sowing (S1,77d) than normal sowing date (S2,66 d), while at HH, days from emergence-flowering for S1 and S2 were similar. At HZ, the effect model of sowing date on development was similar in the two years: days from emergence-flowering prolonged 6-8 d when early sowing and shortened 9-11 d when late sowing; days from flowering to maturity shortened 3-5 d in both cases of early and late sowing. At SD, the days from emergence to flowering were prolonged by 5-7 d when early sowing occurred; 3-7 d were shortened when late sowing occurred; and days from flowering to maturity remained relatively stable. (45-46 d) in 2021; the same days (38 d) occurred when early sowing (S1) and conventional sowing dates (S2) were used but were significantly prolonged to 48 d when late sowing occurred in 2020.

#### 3.5.6 Variety-location-sowing date interaction

**Figure 11:**
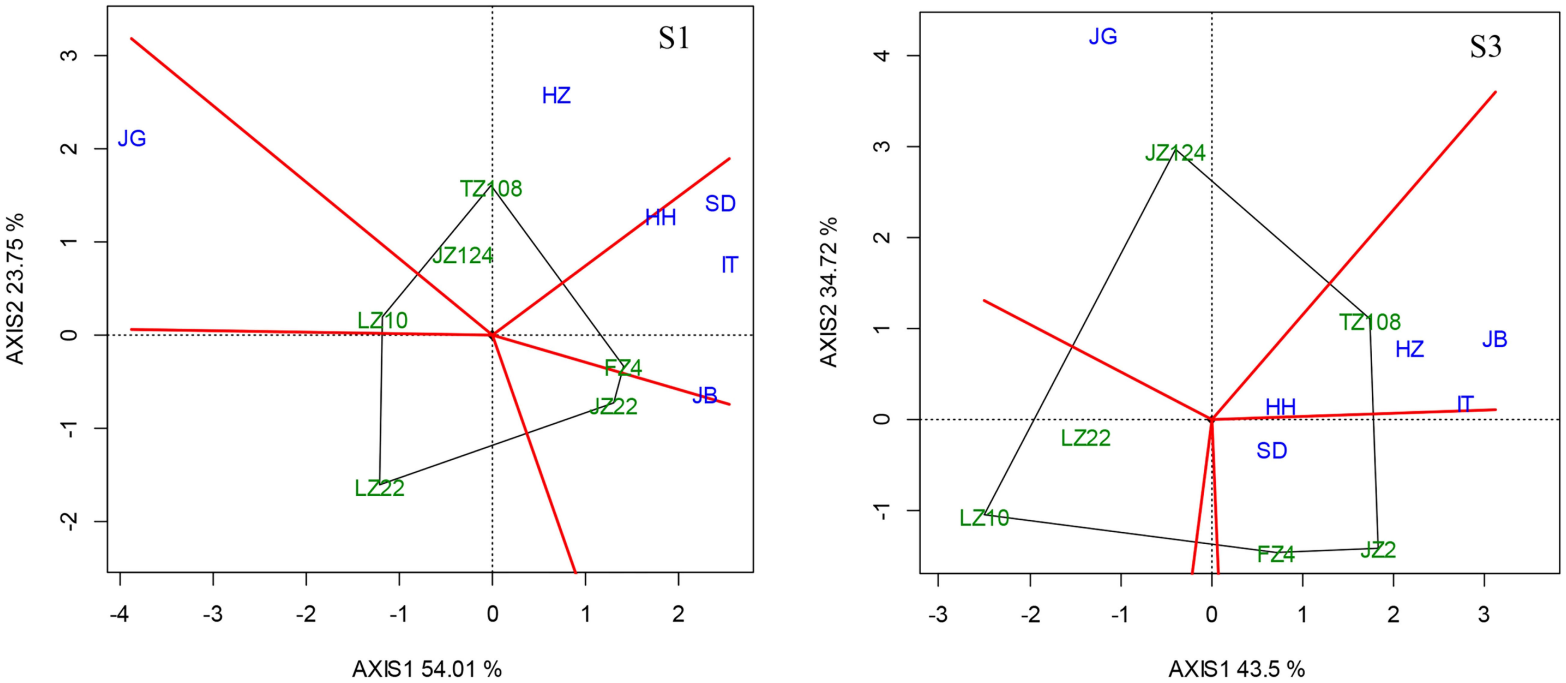
“Which Won Where/What” biplot under different sowing dates (Left, early sowing, S1; Right, late sowing, S3).

This interaction is revealed with the “Which Won Where/What” biplot under early (S1) and later sowing (S3), as showed in **Figure 11** as compared with **Figure 7**, where we can easily find the difference in biplot modes under three sowing dates. In the context of early sowing, 6 locations were clustered into 3 groups or environments, i.e., HH, SD, IT and JB were in a group (Group 1); HZ and JG were respectively in the other two groups (Group 2 and Group 3). In group 1, FZ4 performed best on average. In group 2, JZ124 and TZ108 fit it, and TZ108 was better. In Group 3, LZ 10 outperformed the other locations. In the context of conventional sowing date, six locations were clustered into 3 groups as same as in the context of conventional sowing date. In group 1, HH, SD, IT, and HZ were almost overlapped, and TZ108 was suitable here; JB and JG were respectively in the other two groups (Group 2 and Group 3). FZ4 and JZ22 were more suitable for JB than other varieties, while LZ22 performed better at other locations. In the case of late sowing, HH, IT, HZ, and JB were in Group 1 where TZ108 performed best; SD and JG were respectively in Group 2 and Group3. JZ124 performed best at JG; JZ22 and FZ4 performed better at SD.

## 4 Discussion

### 4.1 Contribution of genotype, environment, management and their interactions

Optimizing genotype-environment-management is an important work to achieve highly efficient production based on understanding the simple effects and interactive effects of genotype, environment, and cultural practices (Owuor et al., 2011; Xin and Tao, 2019; Sellamae et al., 2021) . Although much research has been conducted on related aspects of interaction, systematic interpretation is scarce, and little is understood about the relative contribution of each component (G, E, and M) and their interactions that influence sorghum yield (Ciampitti et al., 2019). In the present research, we interpretated in detail the roles of each factor (year, location, hybrid, sowing dates and plant density) and their interactions in the SSEM areas of China. The interacted relationship of various factors was made clear. We found wide and significant differences existed among varieties, locations, sowing dates, and densities, and wide and significant interactions were also found. Location was the largest contributor to yield variance (38.18%), followed by variety (12.31%), and agronomic practices (sowing date and density, totally 2.07%). This result was similar to Carcedo et al. (2021) research in Pampas of Argentina, in which environment contributed for 35% of yield variance, and genotype and sowing dates contributed for 18% and 5% respectively. As for the contribution of the interactions, our research showed year-location-genotype and location-genotype contributed most to yield variance, 9.02% and 8.07% respectively, while in Carcedo et al. (2021) research, sowing-genotype interaction was the largest contributor, accounted for 19% of the yield variance. In total, our result showed interactions contributed for a larger proportional part of yield variance (31.98%), and it is required to consider all factors if we would like to make full use of natural resources to obtain the highest yield. However, we think that a long period of research is needed until we master the changing law of climate due to the complexion of the relation between the effects of variety and management and changing climatic factors.

### 4.2 G-E-M interactions and changing climate

G-E-M interactions were ultimately caused mainly by changing climatic factors and responses of genotypes. Saeed and Francis (1984) estimated the relative contribution of weather variables during various plant growth stages to variation in environment and genotype-environment interactions of grain sorghum for three growth stages viz., planting to panicle initiation (GS1), panicle initiation to anthesis (GS2), and anthesis to physiological maturity (GS3). The extent to which these variables contributed to differential genotypic response varied among maturity groups and growth stages. The temperature was the most important factor affecting the environmental variability in yield. The effects of temperature and rainfall in GS2 and GS3 were highly associated with GE interaction effects for yield in all maturity groups. In the present research, we analyzed the weather factors during the stage from emergence to anthesis (equivalent to GS1+GS2 minus days to emergence) and the stage from anthesis to physiological maturity (GS3) in relation to the yield under three sowing at six locations and found that climatic factors in GS3 contributed much more proportion to the yield variance. Our research showed that the significant year-sowing interaction in the present research was caused by changed weather conditions over two years. Adjei-Twum (1987) documented that the post-anthesis rainfall was more critical for seed yield than was the seasonal total. As with the improvement of weather forecasting techniques and intelligent techniques, and a deep understanding of the interaction of genotypes, changing climatic factors, and proper agronomic practices, sorghum would be planted at a more suitable sowing window under rainfed conditions.

### 4.3 G-E-M interaction and variety selection

The selection of suitable varieties in a specific location is a very important agronomic practice in crop production, especially in areas with one or more restricted environmental factors. Early maturing varieties could get mature long before frost killing, but natural light and temperature resources could not be fully mined, and the yield of early maturing varieties was generally not as high as that of mid- or late maturing varieties. Generally, a full-season variety is the best choice, although with the risk on early frost killing. In the present research, the early-maturing varieties LZ10 and LZ22 did not outperform mid-late maturing varieties in all sowing contexts, even in the case of late sowing, in which other sorghum varieties did not reach full maturity before frost killing. Therefore, in such areas of high latitude with a short frost-free period, it may be considered to select a full season high yield variety that could get mature in the general case and could develop to the hard dough stage in special years. In a study in Missouri, Conley et al. (2003) had a similar finding, where the early-season hybrids were often lower yielding than the mid- or late-season hybrids, and there was never a yield advantage to planting early-season hybrids even on the latest planting dates.

### 4.4 Timing of sowing in grain sorghum production

To set a suitable sowing window, we need understanding the effect of climatic change on sorghum growth and development, and master the change law of climate, the law of rainfall, and the law of temperature change in a year and among years. For sorghum, the flowering and maturation time is the most important factor that we have to consider when setting a suitable sowing window. The evidence strongly suggests that atmospheric dryness during grain development might have an appreciable influence on grain yield (Stern, 1968). In areas such as Harbin, Tongliao, or Datong, where the frost-free period is short, the temperature is a limiting factor of yield, and sowing as early as possible when soil temperature and moisture meet the basic needs of seed germination is an important practice. The situation of these areas is similar to that in Ukraine, where field experiments in 2013–2015 showed that early sowing led to persistently higher yield compared to late sowing (Domaratsky et al., 2018) . In contrast, in areas such as Zhangjiakou, which has a relatively longer frost-free period, proper delayed sowing may be suitable to obtain a higher yield if sorghum can get mature. However, planting date had a significant effect on early-season seedling emergence and varied between years (Radford and Henzell,1990; Razmi et al., 2013; Zander et al., 2021), climatic characteristics of different years, cold tolerance of variety should be considered for setting a proper early sowing date.

### 4.5 Optimal plant density range

The effect of plant density on yield is widely observed around the world, but few reports on its interaction with other factors. In our present research, only plant density-location and density-year-location were detected to be significant, showing the stability of sorghum grain yield over a wide range of plant densities. In the early years, it was thought that planting in wide rows was advantageous in drylands because within-row plant competition would reduce tillering, vegetative production, and early-season water use, thus, possibly conserving soil water for use during grain filling (Brown and Shrader, 1959; Thomas et al., 1981) . It was later proven that the application of wide rows would result in a considerable sacrifice of grain yield during these favorable growing seasons (Jones & Johnson, 1991; Fernandez et al., 2012) . Higher density and wider row spacing may induce morphological changes rendering plants to log (Gondal et al., 2018). In Adjei-Twum (1987)’s research, within the levels of density tested, there was great variability in grain yield from year to year, and the highest density did not reach the optimum for the area. These results indicated that a proper higher density may be a better choice even in low fertility land. Adverse conditions frequently lead to varied population density in different years and locations, making precision sowing and management a great challenge, so wide adaptation to density seems more important under mechanized production systems.

## 5 Conclusion

Through a two-year field experiment with 6 varieties at 6 locations in the early mature regions of China, we found that the yield difference between years was significant, and the yield differences among locations, varieties, sowing dates, and densities were all highly significant. The variety effect was mainly influenced by environment and sowing dates. The sowing effect was mainly influenced by environment and variety. The plant density effect was only highly influenced by location or location-year interaction. The location was the largest contributor to the total yield variance, followed by variety. Wide interaction existed among variety, year and location, which played an important role in yield formation. The management practice including sowing date and density only contributed for a small part to the yield variation, but it is still important for high yielding. The sowing date significantly affected the growth and development process in sorghum, especially during the late period. The yield was mainly affected by climatic conditions during the late development stage. Keys climatic factors affecting yield varied with locations and years.

## Data availability statement

The original contributions presented in the study are included in the article/**Supplementary Material**. Further inquiries can be directed to the corresponding authors.

## Author contributions

F-CG, data processing and statistical analysis and writing; H-DY, YG, YH, ML, G-LS, Y-MR, and J-HL in charge of field experiments in each location; Y-XJ, Y-JT, Y-XW, TL, G-YF, Z-GW, R-FG, and F-HM, participated in field experiments and data collection; F-XH, S-JJ, and G-YL, experiment design, analysis, writing, and revision. All authors contributed to the article and approved the submitted version.

## Funding

This research is supported in part by the National Key Research & Development Program of China (2019YFD1001700), Science and Technology Innovation Project of the Chinese Academy of Agricultural Sciences.

## Acknowledgments

This research was accomplished by many staff members in the institutions above, and only some of them are listed as the authors. Many thanks to all those who contributed to this research, but not listed.

## Conflict of interest

The authors declare that the research was conducted in the absence of any commercial or financial relationships that could be construed as a potential conflict of interest.

## Publisher’s note

All claims expressed in this article are solely those of the authors and do not necessarily represent those of their affiliated organizations, or those of the publisher, the editors and the reviewers. Any product that may be evaluated in this article, or claim that may be made by its manufacturer, is not guaranteed or endorsed by the publisher.
