# Supplementary material for: Interpretation of genotype-environment-sowing date/plant density interaction in sorghum [Sorghum bicolor (L.) Moench] in early mature regions of China
Source: Front Plant Sci. 2022 Sep 21;13:1008198. doi: 10.3389/fpls.2022.1008198 (PMC9533098; doi:10.3389/fpls.2022.1008198)
Supplement: Supplementary file 1 [file DataSheet_1.docx]

**Supplementary Figures**


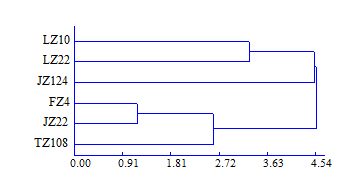

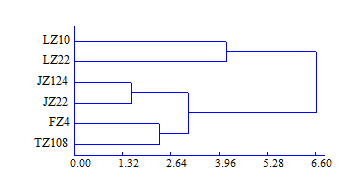


**Supplementary Fig. S1** Clustering of varieties with yield performance in six locations (left, 2020; right, 2021). Six varieties, LZ10, LZ22, JZ124, FZ4, JZ22, TZ108

**Supplementary Fig. S2.** Interaction of variety and year revealed by yield. ‘*’,‘**’mean yield difference between two year was significant at 0.05 and 0.01; ns, not significant.

**Supplementary Fig. S3.** Interaction of location with density and years. ‘*’,‘**’mean yield difference between two year was significant at 0.05 and 0.01; ns, not significant.

**Supplementary Fig. 4.** Development processes of six varieties as affected by the sowing dates in six locations 2020-2021 (to be continued)

**Supplementary Fig. 4.** Development processes of six varieties as affected by the sowing dates in six locations 2020-2021 (Continued)

HH, Harbin; JB, Baicheng; JG, Gongzhuling; IT, Tongliao; HZ, Zhangjiakou; SD, Datong; 20 and 21 is the short form of 2020 and 2021.
