# Supplementary material for: Interpretation of genotype-environment-sowing date/plant density interaction in sorghum [Sorghum bicolor (L.) Moench] in early mature regions of China
Source: Front Plant Sci. 2022 Sep 21;13:1008198. doi: 10.3389/fpls.2022.1008198 (PMC9533098; doi:10.3389/fpls.2022.1008198)
Supplement: Supplementary file 2 [file DataSheet_2.docx]

**Supplementary Table**

**Supplementary Table S1． Information of varieties used in this study**

| Serial Number | Name of variety | Breeding institutions | Breeding location | Main characteristics | Adapted Region |
| --- | --- | --- | --- | --- | --- |
| 1 | Longza 10 (LZ10) | Heilongjiang Academy of Agricultural Sciences | Harbin, Heilongjiang | Dwarf (136 cm), days from emergence to maturity is approximately 115 days. | First accumulated temperature zone in Heilongjiang, Required average accumulated active temperature 2757 ℃. |
| 2 | Longza 22  (LZ22) | Heilongjiang Academy of Agricultural Sciences | Harbin, Heilongjiang | Dwarf (117 cm), days from emergence to maturity is approximately 106 days. | First accumulated temperature zone in Heilongjiang, Required average accumulated active temperature 2561 ℃ |
| 3 | Jiza 124  (JZ124) | Jilin Academy of Agricultural Sciences | Gongzhuling, Jilin | plant height 171 cm; middle and early maturity, the growth period of Jiza 124 is 121 days | Songyuan, Baicheng and Changchun areas of Jilin Province, the first accumulated temperature zone of Heilongjiang Province and the east of Inner Mongolia. Required average accumulated active temperature 2831 ℃ |
| 4 | Fengza 4  (FZ4) | Institute of High-tech Crop Breeding, Gongzhuling National Agricultural Science, and Technology Park | Gongzhuling, Jilin | The plant height is 169m.  The average growth period is 119 days. | Chifeng, Tongliao, and Xing'an League of Inner Mongolia autonomous region, Required average accumulated active temperature 2803 ℃. |
| 5 | Jinza 22  (JZ2) | Shanxi Academy of Agricultural Sciences | Jinzhong, Shanxi | The plant height is 194 cm. The average growth period is 121 days. | Mid- and late maturing area in Shanxi Province. Required average accumulated active temperature 2818 ℃. |
| 6 | Tongza 108  (TZ108) | Tongliao Institute of Agriculture and Animal Husbandry, Inner Mongolia | Tongliao, Inner Mongolia | The plant height is 153 cm; The average growth period is 120 days | the south of Tongliao City and Chifeng City in Inner Mongolia Autonomous Region, Required average accumulated active temperature 2822 ℃. |

**Supplementary Table S2.** Key meteorological information of 6 locations during the growth period in 2020-2021

| Locations | Latitude | Longitude | Year | Dates Sowing -Mature | Accumulative temp. during growth ( ) | Accumulative diff temp | Accumulative sunlight  (hrs) | Accumulative precipitation (mm) | Mean Relative moisture |
| --- | --- | --- | --- | --- | --- | --- | --- | --- | --- |
| HH | 126.62116 | 45.90978 | 2020 | 5.7-9.18 | 2753.3 | 1202.3 | 1473.1 | 692.3 | 77.3 |
|  |  |  | 2021 | 5.13-10.8 | 2989.3 | 1388.7 | 985.3 | 512.0 | 76.3 |
| JB | 122.81182 | 45.62345 | 2020 | 4.30-9.27 | 3066.9 | 1593.3 | 1168.8 | 668.2 | 68.5 |
|  |  |  | 2021 | 4.30-9.26 | 3038.9 | 1487.4 | 1109.9 | 484.3 | 74.9 |
| SD | 113.173897 | 39.901649 | 2020 | 5.2-10.3* | 3172.9 | 1875.1 | 1488.3 | 288.2 | 51.8 |
|  |  |  | 2021 | 4.29-10.10* | 3301.7 | 1944.8 | 1187.1 | 273.6 | 51.2 |
| HZ | 114.92255 | 40.67291 | 2020 | 4.26-10.3 | 3239.2 | 2192.3 | 1423.7 | 447.9 | 62.1 |
|  |  |  | 2021 | 4.25-10.1 | 3207 | 2033.3 | 1148.7 | 296.6 | 62.3 |
| IT | 122.55393 | 43.73599 | 2020 | 4.29-9.22 | 3059.4 | 1682.6 | 1048.1 | 432.9 | 68.3 |
|  |  |  | 2021 | 4.29-10.3* | 3295.4 | 1621.7 | 1043.4 | 417.5 | 64.1 |
| JG | 124.81554 | 43.52090 | 2020 | 5.2-9.30* | 3211.3 | 1473.1 | 1080.7 | 593.3 | 70.4 |
|  |  |  | 2021 | 5.2-9.10 | 2825.4 | 1211.7 | 851.8 | 482.1 | 74.7 |

*Some varieties could not reach full maturity.

**Supplementary Table S3.**  Previous crops and soil nutrients at six experimental locations

| Location | Year | Previous crops | Organic matter content | Total nitrogen | Alkali-hydrolyzed  nitrogen | Available phosphorus | Available potassium | pH |
| --- | --- | --- | --- | --- | --- | --- | --- | --- |
| Baicheng  (JB) | 2020 | Mung bean | 20.7 | 0.7 | 71.4 | 12.4 | 98.3 | 6.9 |
|  | 2021 | Mung bean | 20.2 | 1.1 | 84.3 | 27.3 | 130.7 | 7.1 |
| Datong  (SD) | 2020 | Soybean | 8.6 | 0.4 | 30.8 | 6.7 | 80.7 | 8.4 |
|  | 2021 | Soybean | 10.7 | 0.6 | 34.3 | 19.7 | 158.5 | 8.5 |
| Gongzhuling  (JG) | 2020 | Maize | 24.3 | 1.1 | 97.3 | 22.2 | 140.8 | 6.4 |
|  | 2021 | Maize | 19.3 | 1.3 | 96.9 | 57.3 | 188.5 | 5.3 |
| Harbin  (HH) | 2020 | Soybean | 31.1 | 1.1 | 126.6 | 25.2 | 218.4 | 7.0 |
|  | 2021 | Flax | 26.2 | 1.3 | 106.0 | 21.7 | 179.6 | 7.5 |
| Tongliao  (IT) | 2020 | Soybean | 20.2 | 0.8 | 62.3 | 6.7 | 139.5 | 8.6 |
|  | 2021 | Maize | 13.1 | 0.8 | 49.6 | 7.5 | 97.4 | 8.4 |
| Zhangjiakou  (HZ) | 2020 | Okra | 22.1 | 0.5 | 32.9 | 34.0 | 141.5 | 8.5 |
|  | 2021 | Broomcorn millet | 22.7 | 1.1 | 57.0 | 29.6 | 213.2 | 8.1 |

**Supplementary Tab. S4.** Result of 'Interactive-forward-selection’ of environmental variables based on six locations under three sowings in 2020.

| Name | Explains % | Contribution % | pseudo-*F* | *P* |
| --- | --- | --- | --- | --- |
| EAT-1 | 12.6 | 12.9 | 6.9 | **0.026** |
| TSH-1 | 6.9 | 7 | 2.1 | 0.184 |
| AAT-1 | 3.8 | 3.8 | 1.2 | 0.342 |
| MGT5-1 | 3.7 | 3.8 | 1.1 | 0.300 |
| TRF-1 | 2 | 2.1 | 0.6 | 0.476 |
| MGT10-1 | 1.5 | 1.6 | 0.4 | 0.538 |
| MRH-1 | 1.1 | 1.1 | 0.6 | 0.496 |
| Sub-total | 31.6 | 32.3 |  |  |
| MGT5-2 | 32.1 | 32.8 | 7.5 | **0.012** |
| AAT-2 | 16.8 | 17.2 | 4.9 | **0.030** |
| TRF-2 | 4.9 | 5 | 1.5 | 0.214 |
| EAT-2 | 4.7 | 4.8 | 3.4 | 0.120 |
| TSH-2 | 4.3 | 4.4 | 6.3 | **0.052** |
| MRH-2 | 3 | 3.1 | 0.9 | 0.368 |
| MGT10-2 | 0.5 | 0.5 | 0.6 | 0.560 |
| Sub-total | 66.3 | 67.8 |  |  |

**Supplementary Tab. S5.** Result of 'Interactive-forward-selection’ of environmental variables based on six locations under three sowings in 2021.

|  |  |  |  |  |
| --- | --- | --- | --- | --- |
| Name | Explains % | Contribution % | pseudo-*F* | *P* |
| MGT10-1 | 6.2 | 6.2 | 4.8 | 0.046 |
| MRH-1 | 5.7 | 5.7 | 14.8 | 0.008 |
| EAT-1 | 4.6 | 4.6 | 1.3 | 0.28 |
| AAT-1 | 1.3 | 1.3 | 0.7 | 0.458 |
| TSH-1 | 1 | 1 | 0.5 | 0.524 |
| MGT5-1 | 0.6 | 0.7 | 2 | 0.208 |
| TRF-1 | 0.6 | 0.6 | 2.3 | 0.198 |
| Sub-total | 20 | 20.1 |  |  |
| MGT5-2 | 23.1 | 23.3 | 12.1 | 0.006 |
| TSH-2 | 21.1 | 21.2 | 5.2 | 0.034 |
| MRH-2 | 17.8 | 17.9 | 3.5 | 0.07 |
| TRF-2 | 10.5 | 10.6 | 2.9 | 0.112 |
| EAT-2 | 4.4 | 4.4 | 2.6 | 0.158 |
| AAT-2 | 1.3 | 1.3 | 0.7 | 0.47 |
| MGT10-2 | 1.1 | 1.1 | 0.6 | 0.472 |
| Sub-total | 79.3 | 79.8 |  |  |
